# Stability of the HTLV-1 glycoprotein 46 (gp46) gene in an endemic region of the Brazilian Amazon and the presence of a significant mutation (N93D) in symptomatic patients

**DOI:** 10.1186/s12985-018-0984-9

**Published:** 2018-05-02

**Authors:** Maria de Nazaré do Socorro de Almeida Viana, Akim Felipe Santos Nobre, Edivaldo Costa Jr, Ingrid Christiane Silva, Bruna Teles Pinheiro, Cássia Cristine Costa Pereira, Louise de Souza Canto Ferreira, Danilo Souza de Almeida, Marcos William Leão de Araújo, Mariza da Silva Borges, Carlos Araujo da Costa, Edna Aoba Yassui Ishikawa, Stephen Francis Ferrari, Maísa Silva de Sousa

**Affiliations:** 1 Postgraduate Program in Tropical Diseases, Núcleo de Medicina Tropical, Universidade Federal do Pará, Belém, Pará Brazil; 2 000 0004 0620 4442 Virology Section, Instituto Evandro Chagas, Health Surveillance Secretariat, Brazilian Ministry of Health, Ananindeua, Pará Brazil; 3 Faculty of Pharmacy, Universidade Federal do Pará, Belém, Pará Brazil; 4 Faculty of Nursing, Universidade Federal do Pará, Belém, Pará Brazil; 5 Department of Ecology, Universidade Federal de Sergipe, São Cristóvão, Sergipe Brazil

**Keywords:** Genetic diversity, Molecular evolution, Neglected diseases

## Abstract

**Background:**

The human T-lymphotropic virus type 1 (HTLV-1) affects 2–5 million people worldwide, and is associated with a number of degenerative and infectious diseases. The Envelope glycoproteins (gp) are highly conserved among the different HTLV-1 isolates, although nucleotide substitutions in the region that codifies these proteins may influence both the infectivity and the replication of the virus. The gp46 gene has functional domains which have been associated with the inhibition of the formation of the syncytium, cell-cell transmission, and the production of antibodies. The present study investigated the genetic stability of the gp46 gene of HTLV-1 in an endemic region of Brazilian Amazonia.

**Methods:**

Index case (IC - a sample of a given family group) carriers of HTLV-1 were investigated in the metropolitan region of Belém (Pará, Brazil) between January 2010 (registered retrospectively) and December 2015. The sequences that codify the gp46 were amplified by PCR, purified and sequenced (MF084788–MF084825). The gene was characterized using bioinformatics and Bayesian Inference.

**Results:**

The 40 patients analyzed had a mean age of 45.2 years and 70% presented some type of symptom, with a predominance of pain and sensitivity, dysautonomia, and motor disorders. All patients presented the aA (Transcontinental Cosmopolitan) genotype, with an extremely low mutation rate, which is characteristic of the codifying region (aA – 1.83 × 10–^4^ mutations per site per year). The gp46 gene had a nucleotide diversity of between 0.00% and 2.0%. Amino acid mutations were present in 66.6% of the samples of individuals with signs/symptoms or diseases associated with HTLV-1 (*p* = 0.0091). Of the three most frequent mutations, the previously undescribed N93D mutant was invariably associated with symptomatic cases.

**Conclusions:**

The aA HTLV-1 subtype is predominant in the metropolitan region of Belém and presented a high degree of genetic stability in the codifying region. The rare N93D amino acid mutation may be associated with the clinical manifestations of this viral infection.

**Importance:**

Little is known of the phylogeny of HTLV-1 in the endemic region of Brazilian Amazonia, and few complete gene sequences are available for the gp46 glycoprotein from the local population. The nucleotide sequences of the viral gp46 gene recorded in the present study confirmed the genetic stability of the region, and pointed to a homogeneous viral group, with local geographic characteristics. Further research will be necessary to more fully understand the molecular diversity of this protein, given the potential of this codifying region as a model for an effective HTLV-1 vaccine. The identification of a rare mutation (N93D), present only in symptomatic patients, should also be investigated further as a potential clinical marker.

**Trial registration:**

ISRCTN 12345678, registered 28 September 2014.

**Electronic supplementary material:**

The online version of this article (10.1186/s12985-018-0984-9) contains supplementary material, which is available to authorized users.

## Background

In 1980, the first human retrovirus was found in cells of the T lineage, which was denominated the Human T-cell Lymphotropic Virus (HTLV) [1]. Four variants of this virus are now known in humans, and are referred to as the Human T-cell Lymphotropic Virus, types 1–4 (HTLV-1, HTLV-2, HTLV-3, and HTLV-4). This virus belongs to the genus *Deltaretrovirus*, family Retroviridae [2]. Worldwide, only 2–5% of infected individuals present symptoms, while most are asymptomatic throughout their lives. The current data indicate that there may be 5–10 million carriers of HTLV-1 worldwide, with approximately 1.5 billion individuals living in endemic areas [3]. An estimated 2.5 million individuals are thought to be infected in Brazil [4], and in the Amazon region, infections by both HTLV-1 and HTLV-2 have been recorded in both urban and rural populations, with a certain degree of endemicity [4, 5]. While it may often be asymptomatic, HTLV-1 has been implicated in the development of diseases such as leukemia/Adult T-cell Lymphoma (ATL), HTLV-1 associated myelopathy/Tropical Spastic Paraparesis (HAM/TSP), and a number of other inflammatory diseases, including dermatitis, uveitis, arthritis, and strongyloidiasis [6].

The HTLV-1 genome has three regions that codify the precursors *gag*, *pol* and *env*. The glycoproteins of the env region mediate the binding of the virus to the surface receptors of the target cells. The gp46 surface Envelope glycoprotein is essential to the initial steps of the viral infection, and is the most immunogenic protein of all viral antigens. A large proportion of the neutralizing antibodies are directed towards the Envelope glycoprotein [7]. The reduced genetic diversity of the gp46 sequences is related to the fact that the HTLV-1 genome varies little, in general, given that it persists in an individual throughout the clonal expansion of infected cells. However, a certain amount of variation does exist among geographic regions and in some HTLV-1 subgroups, which may be associated with different levels of vulnerability to disease [8].

Understanding the immunogenic properties of the HTLV-1 surface Envelope glycoprotein will be crucial for the development of effective vaccines and immunological treatments to combat infections [8]. In Brazil, HTLV infection is not treated as a public health problem and is largely neglected, which in general means that the risk of transmission increases substantially [9]. The geographic region investigated in the present study is considered to be an area of endemism for this viral infection, as demonstrated in previous genomic studies [10–12], although the genetic evolution of the virus (which is of paramount importance for the prevention of this pathology) has never been analyzed in this prominent region. The present study is based on the molecular characterization of the gp46 gene of HTLV-1, the analysis of its diversity and evolution, and the identification of the possible factors that determined the changes in its amino acids.

## Methods

### Sample population

A total of 1929 blood (PBMC) samples were collected between January 2010 and December 2015 from patients being monitored for the diagnosis of HTLV, in an outpatients clinic for infectious diseases in Belém, Pará, Brazil. The inclusion criteria for the analysis of the genomic sequences of the *env* (gp46) region were that the patient had been tested for HTLV-1, was recorded in the database, and was classified as an indicator case, that is, the first case discovered and diagnosed in the family or confirmed at the clinic. All patients that agreed to participate, of both sexes, were included in the study. The socio-epidemiological data were obtained from the medical records maintained at the Tropical Medicine Nucleus (TMN) at the Federal University of Pará (UFPA) in Belém.

### Collection and analysis of the samples

A blood sample (approximately 5 ml) was collected from each patient from a peripheral vein directly into a tube containing EDTA, which was stored at 8 °C. The samples were then tested for anti-HTLV antibodies using the Gold ELISA Anti-HTLV 1/2 (REM) kit, following the manufacturer’s instructions. The reactive samples and those with values 20% above or below the cut-off point (suspected cases) were retested for immunoenzymes and proviral DNA.

Viral extraction was also conducted on each sample using the Wizard®Genomic DNA Purification kit, Promega (Madison, Wisconsin, USA), followed by the amplification of the genetic material of the *pX* genomic region using the PCR and nested PCR techniques, and the typing of the HTLV was based on the RFLP polymorphisms, which were identified by the enzymatic extraction of the products of the nested PCRs, using the *Taq* I enzyme [13].

### Amplification of the complete HTLV-1 gp46 glycoprotein

Following the genotyping of the HTLV, and the identification of the infected patients, the complete sequence of the gene for the HTLV-1 p46 glycoprotein was amplified. This was based on three reactions — (i) a PCR with the gp46F1/gp46R1 nucleotide primers, which generated a fragment of 1047 bps, (ii) a semi-nested PCR using the gp46F1/gp46R2 primers, which produced a fragment of 749 bps [8], and (iii) a semi-nested PCR using the gp46F2/gp46R1 primers, which generated a fragment of 627 bps, which complements the fragment amplified in the preceding step (Supplementary Scheme 1).

The PCR for the complete fragment of the gp46 gene was run in 12.5 μL of Go Taq (2×) Green Master Mix, 8.5 μL of water, 10 pmol (0.5 μL) of each primer (gp46F1: 5’CGCCGATCCCAAAGAAAAA3’ and gp46R1: 5’ACATGGAGCCGGTAATCCC3’) and 3 μL (100 ng) of DNA, with a final volume of 25 μL. In the first semi-nested PCR, the same quantity of Go Taq (2×) Green Master Mix was used, together with 10.5 μL of water 10 pmol (0.5 μL) of each primer, that is, gp46F1 and gp46R2 (5’GACGTGCCAAGTGGATAGGC3’), and 1 μL of the amplified DNA, with a final volume of 25 μL. The same amplification conditions were applied, to generate a fragment of 749 bps [8]. In the second semi-nested PCR, 14.0 μL of Go Taq (2×) Green Master Mix was added to 9.2 μL of water, 10 pmol (0.4 μL) of each primer, gp46F2 (5’GCCCCTACTGGAAATTTCAGC3’) and gp46R1, and 1.0 μL of the DNA, for a final volume of 25 μL. A fragment of 627 bps was obtained using the same amplification conditions.

The amplification protocol for all the reactions was based on 30 cycles of 30 s at 94 **°**C for denaturation, 30 s at 55 **°**C for annealing, and 30 s at 72 **°**C for extension, with a final extension of 10 min at 72 **°**C, and then 10 min at 10 **°**C. The PCR products (6 μL) were electrophoresed at 50 mV for 1 h in 1.5% agarose gel.

### Purification of the samples, sequencing and analysis of the data

The pre-sequencing reaction was processed in an automatic thermocycler using a Big Dye® terminator Cycle Sequencing kit (Applied Biosystems). The samples were then purified (in the solution) using the Bigdye Xterminator Purification kit (Applied Biosystems), and the products of the PCR were sequenced in an ABIPrism 3130xl automatic sequencer (Applied Biosystems), based on the dideoxyribonucleotide chain termination method, using an ABI PRISM Big Dye Terminator Cycle Sequencing kit (Applied Biosystems), following the method of [14]. The nucleotide sequences were analyzed and edited in GENEIOUS v.4.8.5 (Biomatters Limited) [15], and aligned with the sequences of other viruses available in GenBank (http://www.ncbi.nlm.nih.gov), using MAFFT v.7 (Katoh Kazutaka) [16]. Sequences of the complete gene (gp46) and those with the largest amount of available information were selected for this analysis.

The phylogenetic trees and distance matrix were constructed using the IqTREE program (Center for Integrative Bioinformatics Vienna) [17], with the trees being constructed using the Maximum Likelihood (ML) approach. A bootstrap analysis, based on 1000 replicates, was used to the confidence of the groupings generated, with the process being repeated 10 times. The trees was displayed in the FigTree software [18].

The genetic diversity among the sequences generated during the study, and comparisons with those obtained from GenBank were obtained in BEAST v. 1.8, with a total of 59 samples being analyzed (containing all the different HTLV-1 subtypes), resulting in the generation of 100 million comparative trees. This analysis was based on Bayesian Inference [19]. Chi-square was used to analyze the differences in the proportions of amino acid alterations between the symptomatic and asymptomatic groups, as well as between the groups with and without known intra-family transmission of the infection. This analysis was run in BioEstat 5.4, considering a significance level of *p* ≤ 0.05.

## Results

### General epidemiological data

During the study period, a total of 1929 samples were tested for HTLV in the NTM/UFPA. The tests identified 51 HTLV-1 positive samples, of which, 40 were classified as indicator cases (ICs) of the families investigated. The 40 indicator samples that provided an adequate amount and good quality DNA were sequenced.

Carriers of HTLV-1 had a mean age of 45.2 years, and 62.5% (25/40) were female. Most (82.5%; 33/40) of the carriers were adults, i.e., of between 19 and 60 years of age, 65.0% (26/40) were married, 47.5% (19/40) had a monthly income of 1–-2 minimum wages, and 52.5% (21/40) had graduated high school (Table 1). All the subjects declared themselves to be heterosexual.

**Table 1:** Socio-epidemiological parameters of the study population

Variable	Number	Percent
Age group		
0–19 years	1	2.5
20–59 years	33	82.5
≥ 60 years	6	15
Marital status		
Divorced	2	5
Married	26	65
Single	11	27.5
Stable union	1	2.5
Average family income		
US$ ≤ 257	7	17.5
US$257–490	19	47.5
US$516–1290	14	35
Educational level		
Illiterate	2	5
Incomplete elementary school	3	7.5
Complete Elementar school	11	27.5
Incomplete middle school	1	2.5
High School graduate	21	52.5
College graduate	2	5

N = absolute number, % = percentage. Source: study protocol, 2017

### Signs and symptoms

Overall, seven (17.5%) of the indicator cases were diagnosed as positive for HAM/TSP. These patients had a mean age of 50 years, and four (57.1%) were female. Twelve (30.0%) of the indicator cases were asymptomatic, while the remaining 21 (52.5%) presented some type of symptom or signs related to the infection during the course of the study. One patient (2.5%) was infected with Hepatitis C, and one other with HIV, and both were receiving clinical treatment. According to the medical records obtained from the TMN/UFPA, the most common symptoms were pain or sensitivity, dysautonomia and motor disorders (Table 2).

**Table 2:** Frequency of the different signs and symptoms of the HTLV-1 indicator cases analyzed in the present study

Signs and symptoms	*N* (%)	Signs and symptoms	*N* (%)
Sensitivity or pain:		Ophthalmology:	
Back pain	1	Headache	3 (7.5%)
Paresthesia	4	Eye discomfort	1
Leg pain	11 (27.5%)	Burning sensation	1
Muscular weakness	10 (25.0%)	Eye pain	1
Arthralgia	6	Clouded vision	1
Cramps		7	
Dysautonomia:		Skin:	
Constipation	6 (15.0%)	Itching	2 (5.0%)
Urinary incontinence	6 (15.0%)	Scaly lesions	1
Frequent urination	2	Circular spots	2 (5.0%)
Erectile dysfunction	1	Furunculosis	1
Motor problems:		Related to infection by the *S. stercoralis* parasite	
Difficulty walking	5	Larvae in the feces	1
Falls	8 (20%)	Anemia	2
		Diarreia	3
		Weight loss (≥10 Kg)	2
		Emaciation	4 (10%)

*Source:* Study protocol, 2017

### Phylogenetic data and analysis of the aminogram

**Fig. 1:**
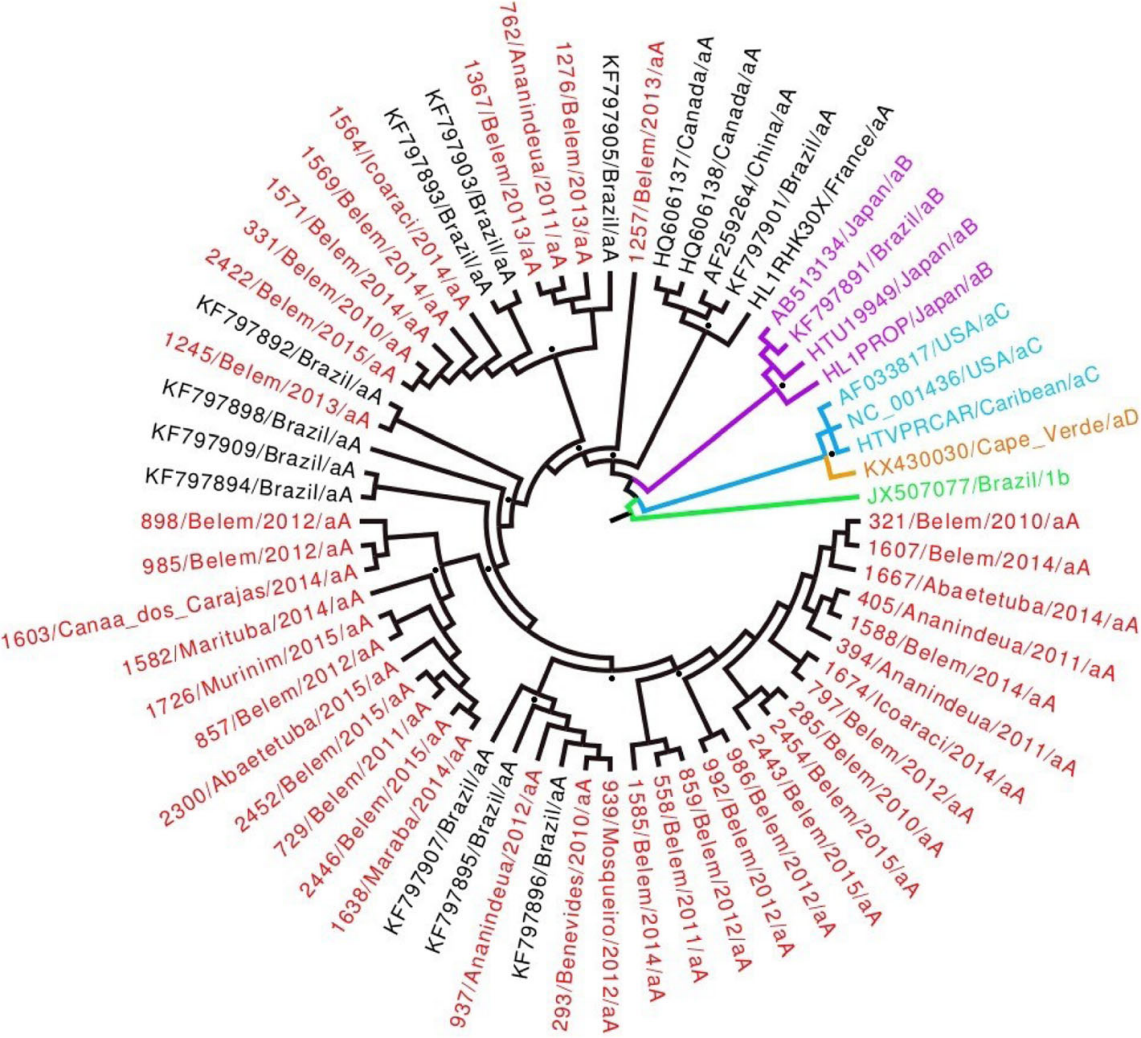
Phylogenetic tree based on the 1047 bp nucleotide sequence of the codifying region of the gp46 gene of the HTLV-1, using the Maximum Likelihood Approach, with 1000 bootstrap replications, repeated 10 times. The clades supported by bootstrap values of at least 70% are marked with a dot (^**.**^) *Source:* Study protocol, 2017

**Fig. 2:**
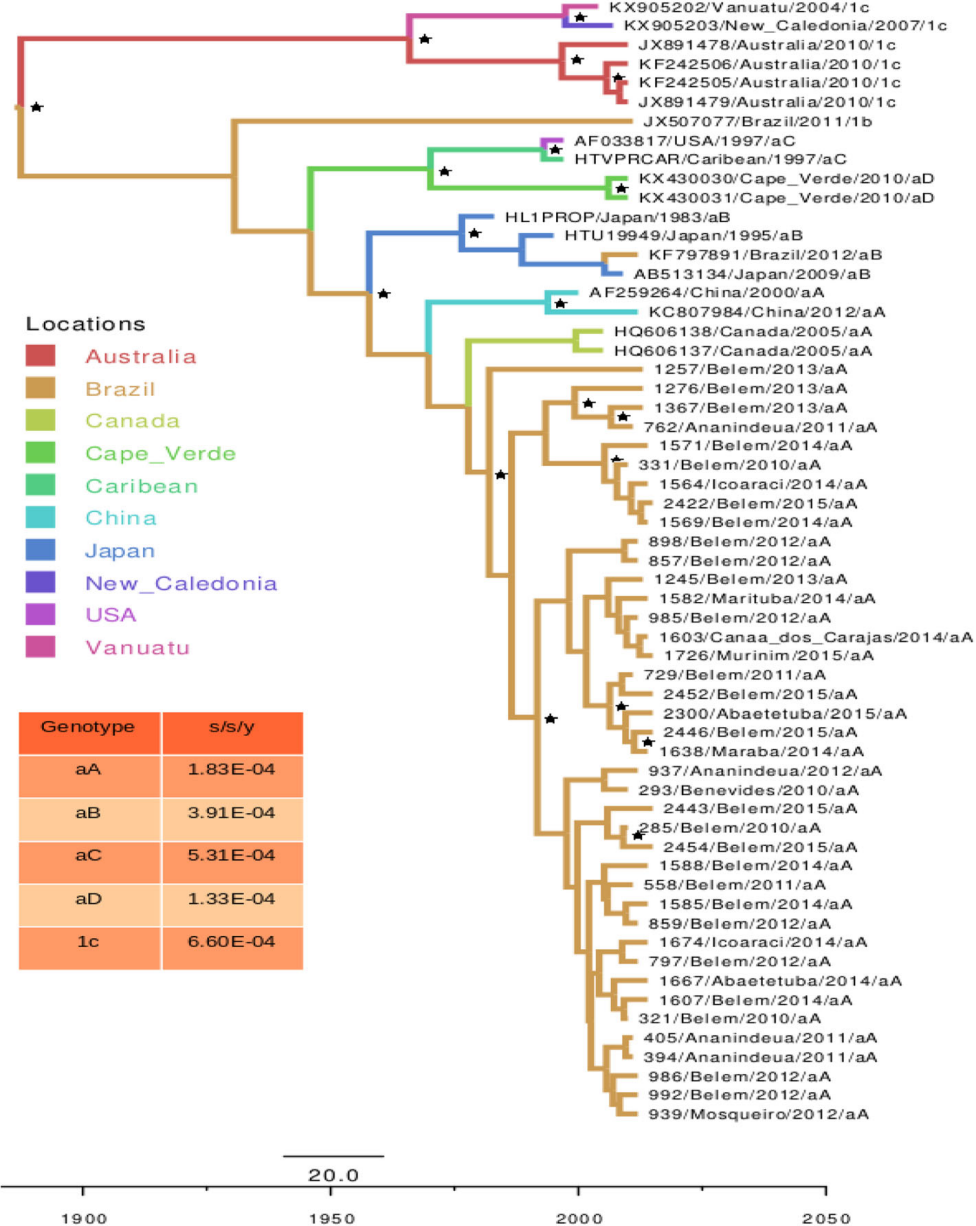
Molecular clock generated by the sequences of the codifying region of the gp46 gene of the HTLV-1, calculated using Bayesian Inference, with 100 million comparative phylogenetic trees, evaluated by the exponential growth method. The clades supported by bootstrap values of at least 70% are marked with an asterisk (*) *Source:* Study protocol, 2017

All the samples presented the HTLV-1 aA (Transcontinental Cosmopolitan) genotype (Fig. 1). The nucleotide diversity of the gp46 gene varied from zero to 2.0% in the 40 indicator samples analyzed in the present study (highlighted in red). Diversity of 0.49–2.59% was recorded in comparison with the four Transcontinental Cosmopolitan samples from other countries. The analysis of genetic diversity (Fig. 2 and Additional file 1: Table S1), which compared 59 HTLV-1 samples, generated 100 million comparative trees, and revealed distinct evolutionary rates for the five subgroups studied, i.e., aA = 1.83 × 10–^4^; aB = 3.91 × 10–^4^; aC = 5.31 × 10–^4^; aD = 1.33 × 10–^4^; 1c = 6.60 × 10–^4^ mutations per site per year.

Just over half (52.5%; 21) of the 40 samples analyzed in this study presented some type of amino acid mutation, with three types – S72G, N93D and S192P – being recorded most frequently (Table 3). The mutations S72G and N93D were each identified in five samples (23.8% of the total). Three of the five individuals with the S72G mutation were symptomatic, one with HAM/TSP and the other two with pain, sensitivity, and dysautonomia. All the cases with the N93D mutation also presented pain, sensitivity, and dysautonomia. The S192P mutation was recorded in four samples (19.0%), of which two were symptomatic (pain, sensitivity, and dysautonomia), and two were asymptomatic.

**Table 3:** Description of the samples that underwent changes in their amino acids, the type of exchange, location of the exchange site, description of the domain and signs / symptoms and / or diseases associated with HTLV-1

Patient	Amino acid mutation of the gp46 gene	Domain/Site	Description of the domain	Signs/Symptoms/Diseases associated with the HTLV-1
2443	**S192P** ^**a**^	175-209aa/ 181-208aa	Region dominated by linear epitopes/ funcional domain	Scaly lesions
285	**S192P** ^**a**^	175-209aa/ 181-208aa	Region dominated by linear epitopes/ funcional domain	Ophthalmological problems, pain, dysautonomia, motor disorder
2454	**S192P**^**a**^**/** S194 T/ L200H/ L210H/ L213P	175-209aa/ 181-208aa/ 197-205aa	Region dominated by linear epitopes/ funcional domain/ funcional domain	Asymptomatic
762	**S192P** ^**a**^	175-209aa/ 181-208aa	Region dominated by linear epitopes/ funcional domain	Asymptomatic
2300	**S72G** ^**a**^	25-190aa/ 53-75aa	Receptor Binding Domain (RBD)/ Region dominated by linear epitopes	Asymptomatic
729	**S72G** ^**a**^	25-190aa/ 53-75aa	Receptor Binding Domain (RBD)/ Region dominated by linear epitopes	Asymptomatic
1638	**S72G** ^**a**^	25-190aa/ 53-75aa	Receptor Binding Domain (RBD)/ Region dominated by linear epitopes	HAM/TSP
2452	**S72G** ^**a**^	25-190aa/ 53-75aa	Receptor Binding Domain (RBD)/ Region dominated by linear epitopes	Sensitivity and pain
558	S35 L	25-190aa	Receptor Binding Domain (RBD)	Asymptomatic
1569	**N93D** ^**a**^	25-190aa/ 53-75aa/ 75-101aa/ 86-107aa/ 90-94aa	Receptor Binding Domain (RBD)/ Region dominated by linear epitopes/ funcional domain/Region dominated by linear epitopes/Amino acid associated with neuropilin 1.	Sensitivity and pain
2422	**N93D** ^**a**^	25-190aa/ 53-75aa/ 75-101aa/ 86-107aa/ 90-94aa	Receptor Binding Domain (RBD)/ Region dominated by linear epitopes/ funcional domain/Region dominated by linear epitopes/Amino acid associated with neuropilin 1.	Sensitivity and pain
331	**N93D** ^**a**^	25-190aa/ 53-75aa/ 75-101aa/ 86-107aa/ 90-94aa	Receptor Binding Domain (RBD)/ Region dominated by linear epitopes/ funcional domain/Region dominated by linear epitopes/Amino acid associated with neuropilin 1.	Sensitivity and pain
1564	**N93D** ^**a**^	25-190aa/ 53-75aa/ 75-101aa/ 86-107aa/ 90-94aa	Receptor Binding Domain (RBD)/ Region dominated by linear epitopes/ funcional domain/Region dominated by linear epitopes/Amino acid associated with neuropilin 1.	Sensitivity and pain
1571	**N93D** ^**a**^	25-190aa/ 53-75aa/ 75-101aa/ 86-107aa/ 90-94aa	Receptor Binding Domain (RBD)/ Region dominated by linear epitopes/ funcional domain/Region dominated by linear epitopes/Amino acid associated with neuropilin 1.	Sensitivity, pain, infection with the parasite *S. stercoralis*/HIV+
293	L163I	25-190aa	Receptor Binding Domain (RBD)	Sensitivity, pain, and motor disorder
1588	S38 N	25-190aa	Receptor Binding Domain (RBD)	Sensitivity and pain
2446	C26S/ **S72G**^**a**^	25-190aa/ 53-75aa	Receptor Binding Domain (RBD)/ Region dominated by linear epitopes	Sensitivity, pain, dysautonomia, motor disorder, infection by *S. stercoralis*
937	L70I/ S103P	25-190aa/ 53-75aa/ 86-107aa	Receptor Binding Domain (RBD)/ Region dominated by linear epitopes/Region dominated by linear epitopes	HAM/TSP
1276	I150V/ L219I	25-190aa	Receptor Binding Domain (RBD)	Sensitivity and pain
857	F14S	Location not described	Domain not described	Asymptomatic
1367	N24H	Location not described	Domain not described	Asymptomatic

(^a^: The amino acid changes with the highest frequencies in the samples) *Source:* Study protocol, 2017.

Overall, a third (7/21) of the individuals with some type of amino acid mutation were asymptomatic, while the other two-thirds presented some symptom or disease associated with the presence of HTLV-1, a highly significant difference (*p* = 0.0091). The most common symptoms were pain, sensitivity, and dysautonomia (pain in the legs, knees, and hands, paresthesia in the hands, difficulty walking, lombalgia). Less common symptoms included urinary incontinence, dermatological lesions, and parasitosis. Almost half (47.3%; 9/19) of the samples that presented no amino acid mutation were asymptomatic, with no significant difference in comparison with the symptomatic group.

Six of the 40 samples analyzed in the present study were diagnosed with HAM/TSP, of which, two presented amino acid mutations (one patient with S72G and the other with both L70I and S103P). Four individuals (2446, 937, 2454, and 1276) presented more than one amino acid mutation in the gp46 sequence. One of these individuals was asymptomatic, one had HAM/TSP, and the two others presented oligosymptoms, which may be associated with HAM/TSP. While asymptomatic, subject 2454 presented the most amino acid mutations of any individual, with five (S192P, S194 T, L200H, L210H, and L213P).

Of the total number of cases, eleven presented familial transmission and seven (63%) presented some amino acid change in the glycoprotein gp46 gene, as against 48% (14/29) who did not present any familial transmission episode but had some amino acid change in the gp46 gene (*p* = 0.6).

## Discussion

The Tropical Medicine Nucleus is renowned as a center of excellence for the treatment and monitoring of HTLV patients in northern Brazil, with more than 90% of its patients being resident in the metropolitan region of Belém. Between 2010 and 2015, 1929 samples of blood were analyzed, of which, 51 tested positive for HTLV-1. While these figures may not be representative of the prevalence of the virus in the Brazilian Amazon region, they may be consistent with the infection rates found in the metropolitan region of Belém.

The phylogeny of the p46 gene revealed that the aA HTLV-1 genotype is found in the study region, which is consistent with the data on blood donors from the state of Pará [20], and the predominance of this subtype in other regions of Brazil, in both patients with associated diseases [21] and asymptomatic individuals [22]. Gessain and Cassar [3] highlighted the role of migration in the dispersal of the aA subtype to many countries and populations. The slave trade between the fifteenth and nineteenth centuries may have played a determining role in the prevalence of the virus in Brazilian populations of African descent [23].

In the phylogenetic tree, the positive HTLV-1 samples were grouped in the aA clade, together with samples from Canada, China, France, and other regions of Brazil. Limitations of the data prevented comparisons with many samples, due to the lack of complete p46 sequences. Most of the samples analyzed are closely-related, reflecting the conservation of the virus, despite the fact that the individuals sampled were indicator cases, and not related, which reconfirms the known lack of genetic diversity in this virus [24, 25]. No other HTLV-1 subtypes were recorded in the present study, which is consistent with limited occurrence of other genotypes, such as subtypes d, e, and f, which are isolated in Africa, the Congo, and Gabon, respectively [26].

This region is likely to suffer greater selection pressure than others, due to its role in the expression of viral bonding proteins, reinforcing the conclusion that peptides derived from the the gp46 Envelope glycoprotein are strong candidates for the development of an effective vaccine [8].

The HTLV-1 subtypes compared in the evolutionary tree also revealed relatively low levels of divergence. In the case of the nucleotide diversity, Wolfe et al. (2005) [27] recorded a rate of 1% for the complete HTLV-1 genome, while a more specific analysis of the *env* region [28] revealed rates of 7.8–8.0%. While the nucleotide diversity found in the present study was intermediate between these extremes, the analyses were limited by a lack of data on complete p46 sequences.

Approximately two-thirds of the patients analyzed in the present study were symptomatic, a rate similar to that recorded in the endemic region of Salvador, Brazil, where 84.3% of patients reported some type of symptom, in particular pain, during their treatment [29]. This relatively high rate may be related to the nature of the subset of patients treated in the outpatients clinic of the TMN/UFPA, which includes family cases, screened blood donors, and other individuals under investigation. The presence of persistent symptoms may also be related to the mean age of the patients (45.2 years), given the potential role of this factor in the occurrence of symptoms related to infection by HTLV-1, in particular pain [30].

These factors may also have contributed to the relatively high incidence of HAM/TSP (17.5%), in comparison with populations from the Caribbean, where the incidence was 1–5% [31], and other populations, with rates of around 5% [32, 33]. Two of the six patients with HAM/TSP presented amino acid mutations, one with S72G and the other with L70I and S103P. Mota-Miranda et al. (2013) [8] recorded the S72G, N42H and F14S mutations in patients with HAM/TSP. In all cases, there was some alteration of the amino acid sequence of the p46 gene.

In the present study, 21 of the samples presented amino acid mutations, of which, S35 L, F14S, and S72G have been described previously, and most are associated with specific functional domains [8]. The most common mutations were S72G, N93D and S192P, and, of these, N93D was the most relevant, given that all the samples with this mutation were associated with similar symptoms of pain and sensitivity. While nothing is known of this mutation, it may be linked to motor symptoms and possibly even HAM/TSP, and may be restricted to the region of the present study. This mutation involves a large segment of the sequence of the p46 gene (25-190aa/53-75aa/75-101aa/86-107aa/90-94aa), which may cause alterations to the Receptor Binding Domain (RBD), the predominant region of the linear epitopes, the functional domain, and the amino acids associated with the interactions with neuropilin 1.

The S192P mutation, while relatively frequent, could not be linked systematically to symptoms, given that two patients were asymptomatic. While it was also among the most frequent mutations, S72G was also found in an individual with more than one amino acid mutation in the sequence of the gp46 gene. This mutation has been associated with patients with HAM/TSP and has also been found in samples from Gabon, Martinique, and Guadaloupe [8]. In this case, it seems reasonable to suggest an association with the development of motor symptoms and/or HAM/TSP. In some protein domains, distinct epitopes have been identified in asymptomatic individuals and patients with HAM/TSP. The latter tend to have a larger set of *env* epitopes in comparison with the asymptomatic individuals, indicating that this diversity affects the cytotoxicity of the CD8-positive T cells, and may be related to the hyper-immune response in individuals with HAM/TSP [34].

Subject 2454 presented the most amino acid mutations in its sequence, with five (S192P, S194 T, L200H, L210H, and L213P), but nevertheless remained asymptomatic throughout the study period, which suggests that these changes did not influence the symptomatology of the patient. None of these mutations has been described previously, and while they affect some domains with well-defined functions, it seems likely that, in this case, other factors that determine the infection are more influential.

Considering that the gp46 glycoprotein is involved directly in the mechanism of adsorption of the virus by the receptor cell, and thus in the transmission of the virus [35], many of the domains presents in the sequence of the gene have a direct influence on the function of this mechanism, which implies that mutations in this sequence may favor or hinder the transmission of the virus. In 63% of the cases in which family transmission of HTLV-1 was observed, some amino acid mutation was also observed, although there was no significant difference in comparison with the individuals in which no family transmission was recorded. This emphasizes the need for a larger sample size for the more systematic evaluation of the relationship between these mutations and the transmission of the virus. Once the most relevant protein domains are located, it may be possible to define clinical markers for the diagnosis of the disease.

While the HTLV-1 genome varies little, the amino acid mutations in the gp46 gene may modify the structure or antigenicity of the principal neutralizing epitopes. These modifications may have a direct effect on the efficacy of the neutralizing antibodies, and may be related to the clinical manifestations, dissemination, and pro-viral charge [36]. The identification of the *env* epitopes responsible for the activation of the immunological system may also be useful for the development of a vaccine [8].

Modifications of the codifying region for *env* are not well documented, and there is little evidence on their importance for the clinical symptoms or the characteristics of the infection. Further research will be important for the understanding of the functional impact of these modifications and their possible association with specific clinical symptoms, and the identification of variations in the host. This is especially important due to the novel nature of the most common mutations.

The present study reinforces the findings of previous research, which have emphasized the low rates of evolution off the gp46 gene, which supports the use of this region as a target for the development of a vaccine. Up to now, no effective treatment has been developed for the HTLV-1 infection, and the molecular investigation of the *env* region, in particular the gp46 glycoprotein, may provide important insights for the development of an effective vaccine.

## Conclusions

A single HTLV-1 subtype (aA) was recorded in the metropolitan region of Belém. The codifying region of the gp46 gene was highly stable, with a low rate of evolution. There was a predominance of certain symptoms, such as pain and sensitivity, dysautonomia and motor disorders, and HAM/TSP was common in infected patients. Amino acid mutations were related to some symptoms, but not to any greater probability of family transmission of the infection. The rare mutation N93D was found invariably in patients with oligosymptoms associated with HAM/TSP.

## Additional file

Additional file 1: The research ethics committee. Source: Study protocol, 2015, **Table S1**. Description of values of AICM applied in the statistics (Bayesian inference) in the study data. Source: Study protocol, 2016. (PDF 1549 kb)

## Abbreviations

ATL: Leukemia/adult T-cell lymphoma
EDTA: Ethylenediamine tetra acetic acid
ELISA: Enzyme-linked immunosorbent assay
GP-46: Glycoprotein-46
GPS: Glycoproteins
HAM/TSP: HTLV-1 associated myelopathy/tropical spastic paraparesis
HTLV: Human T-lymphotropic virus
ML: Maximum likelihood
PBMC: Periferic blood mononuclear cells
RBD: Receptor binding domain
TMN: Tropical medicine nucleus
UFPA: Federal University of Pará
