# Supplementary material for: Stability of the HTLV-1 glycoprotein 46 (gp46) gene in an endemic region of the Brazilian Amazon and the presence of a significant mutation (N93D) in symptomatic patients
Source: Virol J. 2018 May 2;15:80. doi: 10.1186/s12985-018-0984-9 (PMC5930498; doi:10.1186/s12985-018-0984-9)
Supplement: Supplementary file 1 — The research ethics committee. Source: Study protocol, 2015, Table S1. Description of values of AICM applied in the statistics (Bayesian inference) in the study data. Source: Study protocol, 2016. (PDF 1549 kb) [file 12985_2018_984_MOESM1_ESM.pdf]

ETHICS APPROVAL AND INFORMED CONSENT FOR PARTICIPATION

NÚCLEO DE MEDICINA TROPICAL-NMT/ UNIVERSIDADE FEDERAL DO

Plataforma Brasil

PARECER CONSUBSTANCIADO DO CEP

DADOS DO PROJETO DE PESQUISA

Título da Pesquisa: Diversidade Genética dos Vírus Linfotrópicos de Células T do tipo 1 na Região Metropolitana de Belém.

Pesquisador: AKIM FELIPE SANTOS NOBRE

Área Temática:

Versão: 5

CAAE: 31014114.2.0000.5172

Instituição Proponente: Núcleo de Medicina Tropical-NMT/ Universidade Federal do Pará - UFPA

Patrocinador Principal: Núcleo de Medicina Tropical-NMT/ Universidade Federal do Pará - UFPA
CNPQ

DADOS DO PARECER

Número do Parecer: 992.573

Data da Relatoria: 20/03/2015

Apresentação do Projeto:

Trata-se de um projeto de dissertação de mestrado, do tipo transversal, o qual visa investigar a diversidade genética dos retrovírus HTLV-1 circulantes na região metropolitana de Belém, através da avaliação da frequência e a diversidade de genótipos. A introdução do protocolo descreve claramente a problemática do objeto de estudo e a justificativa para a sua execução está embasada na existência de poucos trabalhos que tenham

abordado esta temática na região amazônica, reforçando a necessidade de estudos mais abrangentes e representativos da população infectada para subsidiar pesquisas com possíveis vacinas e antivirais antiHTLV-1. Assim, é declarada a importância de se investigar a epidemiologia e variabilidade genética do HTLV-1 na região metropolitana de Belém, a maior concentração populacional da Região norte do Brasil.

Objetivo da Pesquisa:

Descrição realizada de forma clara, sendo determinado:

1) Objetivo geral: Verificar a diversidade genética dos HTLV-1 circulantes na região metropolitana de Belém.

Endereço: Av. Generalíssimo Deodoro, 92
Bairro: Umarizal **CEP:** 66.055-240
UF: PA **Município:** BELEM
Telefone: (91)3201-0961 **E-mail:** cepbel@ufpa.br

2) Objetivos específicos: (i) Descrever a frequência dos genótipos de HTLV-1; (ii) Determinar a taxa de diversidade entre os genótipos de HTLV-1.

Avaliação dos Riscos e Benefícios:

Riscos:

Os riscos foram apresentados no protocolo de pesquisa e são concernentes à coleta de sangue e a confidencialidade dos dados dos participantes, pois os pacientes serão submetidos à punção sanguínea venosa e também fornecerão informações pessoais para o levantamento dos dados. Foi deixado claro que a equipe de suporte laboratorial possui grande experiência no tema, o que diminui a chance de acidentes, e também foi informado que serão tomadas medidas de guarda dos dados de pesquisa.

Benefícios:

Foram apresentados benefícios indiretos aos participantes, sendo descritos como a geração de conhecimento sobre a diversidade de tipos virais circulantes, de forma a se propor alternativas profiláticas bem como realizar o controle da doença.

Comentários e Considerações sobre a Pesquisa:

O público alvo do estudo será o de pacientes que foram atendidos no NMT/UFPA durante o período de janeiro de 2010 a dezembro de 2013, da qual participarão da pesquisa aqueles que aceitarem assinar o TCLE e que não possuam amostras inadequadas (coaguladas ou hemolisadas) para analise, sendo esperado um n de 50 pessoas. Serão utilizadas as informações contidas nos prontuários dos respectivos pacientes que aceitarem participar da pesquisa.

Parte das análises do estudo serão realizados no próprio Núcleo de Medicina Tropical e parte no Instituto Evandro Chagas, como apresentado pela anuência em anexo.

Considerações sobre os Termos de apresentação obrigatória:

Todos os documentos foram apresentados e estão de acordo com a resolução 466/12. Estes são:

1) TCLE; 2) Projeto detalhado; 3) Folha de rosto; 4) Parecer de co-participação do Instituto Evandro Chagas.

Recomendações:

Conclusões ou Pendências e Lista de Inadequações:

Como pendências passadas, tinha-se:

1) Na relatoria passada foi solicitado que fosse inserido o Instituto Evandro Chagas como co-participante;

2) Foi também identificado a necessidade de revisão da indicação da área temática, a qual não estava de acordo com os objetivos do trabalho.

Tais solicitação foram acatadas. Deste modo, a situação do parecer é aprovado.

Situação do Parecer:

Aprovado

Necessita Apreciação da CONEP:

Não

Considerações Finais a critério do CEP:

BELEM, 20 de Março de 2015

Assinado por:
ANDERSON RAIOL RODRIGUES
(Coordenador)

ANEXO I

Termo de Consentimento Livre e Esclarecido-TCLE

Título: Diversidade genética do *Vírus Linfotrópico de células T humanas* do tipo 1 na região metropolitana de Belém

O texto abaixo busca prestar esclarecimentos sobre o estudo envolvendo pacientes atendidos no Núcleo de Medicina Tropical.

O *Vírus Linfotrópico de células T humanas 1 e 2* (HTLV 1 e 2) pode provocar doenças no sistema nervoso que podem levar a paralisia nas pernas, impotência, perdas no controle urinário entre outros sintomas, alem de estar associado a casos de Leucemia/Linfoma (tipo raro de câncer). A transmissão familiar se dá através do contato sexual (homem e mulher) ou aleitamento materno (da mãe para o filho). Esta pesquisa tem como objetivo avaliar a transmissão familiar do HTLV em pacientes e seus familiares atendidos no Núcleo de Medicina Tropical (NMT).

Caso você aceite participar, será coletado um pouco de sangue por profissionais treinados para este tipo de trabalho, para que não ocorra nenhum problema durante a coleta.

Posteriormente, nessa amostra de sangue, será investigada a presença do vírus, que será realizada em laboratório especializado da Universidade Federal do Pará (UFPA). Os exames realizados pela pesquisa serão gratuitos, não necessitando nenhum custo por parte do sujeito da pesquisa.

Os casos positivos para a infecção serão orientados e convidados para o acompanhamento clínico-ambulatorial e laboratorial no Núcleo de Medicina Tropical/UFPA, onde serão esclarecidos sobre as formas de se evitar a transmissão do HTLV e sobre as principais doenças relacionadas.

Todas as informações coletadas serão mantidas em sigilo, assegurando o anonimato dos participantes, para evitar qualquer dano moral, uma vez que serão utilizados apenas pelos pesquisadores.

Fica claro que o sujeito da pesquisa ou o seu representante legal, pode a qualquer momento, retirar seu consentimento, que esta pesquisa não apresenta nenhum tipo de ônus para o sujeito da pesquisa e que sua desistência em nada comprometerá o atendimento deste sujeito aos serviços oferecidos pela equipe do projeto.

Consentimento:

Declaro que li e compreendi as informações sobre a pesquisa, que me sinto perfeitamente esclarecido sobre o conteúdo da mesma, assim como os seus riscos e benefícios. Declaro ainda que, por minha livre vontade, aceito participar da pesquisa cooperando com a coleta de material para exame.

Belém, / / 2010.

____

Assinatura do sujeito da pesquisa ou responsável

Akim Felipe Santos Nobre
(Pesquisador responsável)
Endereço: Cidade Nova IX WE 4B, nº 71- Ananindeua–PA; Fone: (91) 81495864; 32352400
Ambulatório do Núcleo de Medicina Tropical: Av. Generalíssimo Deodoro, nº 92, Umarizal; Fone:32016812.

Belém / / 2010

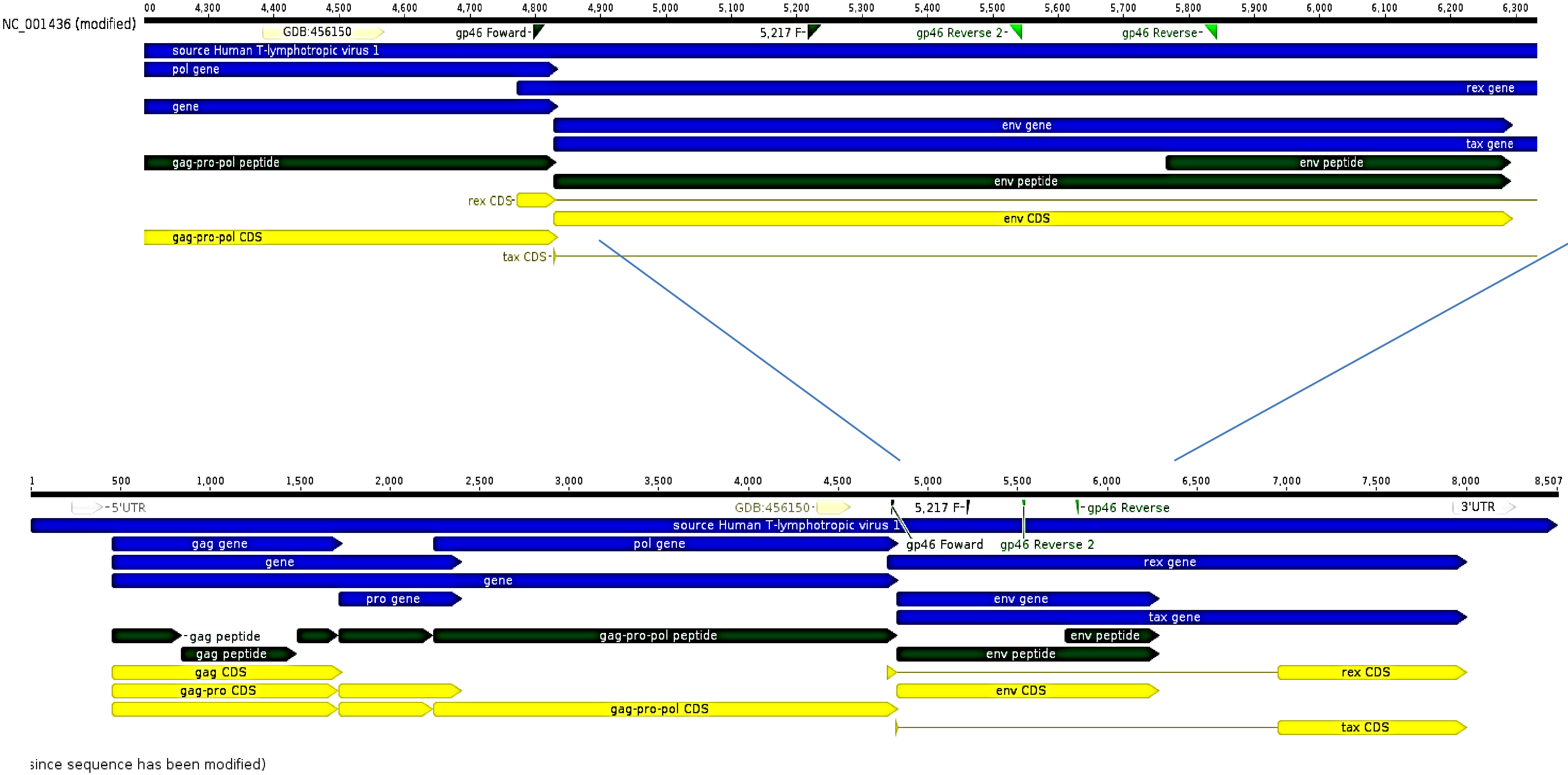

Scheme 1: Localization of the nucleotide primers in the sequence of the gp46 glycoprotein gene, used in the PCR and semi-Nested-PCR reactions, location and size of the fragments generated. Source: Study protocol, 2016

Table 1.: Values of AICM for model selection

Trace	AICM	S.E.	Exponential relaxed clock	Lognormal relaxed clock	Strict Clock
Exponential relaxed clock	8284.129	+/- 0.34	-	130.176	38.067
Lognormal relaxed clock	8414.305	+/- 0.59	-130.176	-	-92.108
Strict Clock	8322.196	+/- 0.308	-38.067	92.108	-

Trace	AICM	S.E.	Exponential Bayesian Skyline	Exponential_constant_size	Exponential_exponential_growth
Exponential Bayesian Skyline	8286.841	+/- 0.32	-	8.209	-7.53
Exponential_constant_size	8295.05	+/- 0.53	-8.209	-	-15.739
Exponential_exponential_growth	8279.311	+/- 0.519	7.53	15.739	-
Exponential_expansion_growth	8401.785	+/- 0.547	-114.944	-106.735	-122.474
Exponential_extended_skyline_plot	8303.306	+/- 0.422	-16.465	-8.256	-23.994
Exponential_logistic_growth	8309.668	+/- 0.245	-22.827	-14.618	-30.357
Exponential_Bayesian_Skygride	8322.62	+/- 0.561	-35.779	-27.57	-43.309
Exponential_GMRF_Bayesian_Skyride	8289.205	+/- 0.194	-2.364	5.845	-9.893

10 Millions of generations

Table 1.: Values of AICM for model selection

Trace
Exponential relaxed clock
Lognormal relaxed clock
Strict Clock

Trace	Exponential_expansion_growth	Exponential_extended_skyline_plot	Exponential_logistic_growth.log
Exponential Bayesian Skyline	114.944	16.465	22.827
Exponential_constant_size	106.735	8.256	14.618
Exponential_exponential_growth	122.474	23.994	30.357
Exponential_expansion_growth	-	-98.48	-92.117
Exponential_extended_skyline_plot	98.48	-	6.362
Exponential_logistic_growth	92.117	-6.362	-
Exponential_Bayesian_Skygride	79.165	-19.315	-12.952
Exponential_GMRF_Bayesian_Skyride	112.581	14.101	20.463

10 Millions of generations

Table 1.: Values of AICM for model selection

Trace
Exponential relaxed clock
Lognormal relaxed clock
Strict Clock

Trace	Exponential_Bayesian_Skygride	Exponential_GMRF_Bayesian_Skyride
Exponential Bayesian Skyline	35.779	2.364
Exponential_constant_size	27.57	-5.845
Exponential_exponential_growth	43.309	9.893
Exponential_expansion_growth	-79.165	-112.581
Exponential_extended_skyline_plot	19.315	-14.101
Exponential_logistic_growth	12.952	-20.463
Exponential_Bayesian_Skygride	-	-33.415
Exponential_GMRF_Bayesian_Skyride	33.415	-

10 Millions of generations
